# Understanding regeneration at different scales

**DOI:** 10.7554/eLife.46569

**Published:** 2019-03-13

**Authors:** Kate MacCord, Jane Maienschein

**Affiliations:** 1 McDonnell InitiativeMarine Biological LaboratoryWoods HoleUnited States; 2 Center for Biology and SocietyArizona State UniversityTempeUnited States

**Keywords:** history of science, philosophy of biology, regeneration, microbial communities, ecosystem renewal, repair

## Abstract

Regeneration occurs at many different levels in nature, from individual organisms (notably earthworms and hydra), through communities of microbes, to ecosystems such as forests. Researchers in the life sciences and the history and philosophy of science are collaborating to explore how the processes of repair and recovery observed at these different scales are related.

The study of regeneration started in the 18th century, following the discovery that some organisms had the ability to regrow body parts that were either damaged or lost. At first, regeneration was studied at the level of single organisms, but in the 1970s, ecologists also began to talk about the regeneration of ecosystems. At first, these discussions centered around the repair of damaged ecosystems, but later the focus shifted to helping the ecosystem to become resilient to change. More recently, it has been proposed that ecosystems should go through a developmental process like individual organisms do in order to regenerate.

In the past decade or so, microbiologists have started to recognize that microbes form dynamic living systems that interact with and within their hosts. The communities of microbes that colonize the human gut and other environments can undergo regeneration after the damage caused by, for example, antibiotics (Antonopoulos et al., 2009). In this context, regeneration has an impact on the microbial community itself and also on the organism that hosts it (Sekirov et al., 2010).

At each of these levels – microbial community, individual organism, ecosystem – we see evidence for a built-in regenerative process that attempts to maintain the integrity of the system by restoring its structure and/or function after damage. Exploring the similarities and differences between the regeneration observed at these different levels is an active area of research in the philosophy of biology at present. For example, we are part of an international network of researchers from the life sciences and the history and philosophy of science, funded by the McDonnell Initiative at the Marine Biological Laboratory in Woods Hole, who are working on this topic. In particular, we are interested in the following questions: What are the basic units and mechanisms involved in the detection and repair of damage at each of the levels? Is there an underlying logic of regeneration that we can recognize across the scales of living systems? If so, how can we apply what we know about one level to the other levels? And if not, are there any relationships between the forms of regeneration observed at each level?

The parallels in systemic responses to injury or stress among individual organisms, microbial communities and ecosystems indicate that regeneration may be a shared phenomenon. However, before we attempt to develop an overarching understanding or theory of regeneration, we first need to understand what is known about regeneration at the level of individual organisms, ecosystems and microbial communities.

## A brief history of regeneration in individual organisms

The idea of regeneration came to the fore in the 18th century through the work of the naturalist Abraham Trembley, who wanted to know why and how the heads of hydra and earthworms could grow back after they had been removed. By the early 20th century, it had been established that many individual organisms were capable of regeneration, and there were three competing explanations of the process: it was due to internal mechanical interactions; it was due to a 'vital force'; or it was an evolutionary effect.

Is there an underlying logic of regeneration that we can recognize across the scales of living systems and if so, how can we apply what we know about one level to the other levels?

Wilhelm Roux, the German zoologist, championed the first of these explanations, based on his studies of frogs. Roux killed cells in early-stage frog embryos and discovered that the frogs continued to develop, but without those body parts that the destroyed cells would have become. For Roux, all life was mechanical, and the fate of each cell in the embryo was, after a few rounds of cell division, determined by properties and mechanisms specific to each cell. In this view, what happened during regeneration was essentially the same as what happened during normal development.

In contrast, Hans Driesch, a German biologist and philosopher, and a contemporary of Roux, conducted similar experiments in sea urchins and reported evidence that challenged Roux's interpretation. Instead of destroying cells, Driesch dissected early-stage sea urchin embryos and showed that new embryos grew from the cells he had removed from the original embryos. Driesch interpreted these results as evidence for the presence of a 'vital force' that was responsible for the development of each embryo. In more modern terms, we could say that Driesch favored a regulatory explanation of development, wherein the fate of a cell was determined by its relationship to the whole organism. In this model, regeneration occurs because the entity informs one or more of its parts that they need to respond to damage.

August Weismann, a German evolutionary biologist best known for his germ plasm theory of heredity, offered a third theory: the fate of a cell was determined by the genetic information contained in its chromosomes, but cells also adapted to environmental pressures. Weismann predicted that the parts most likely to be damaged, like the claws of a crab or the tentacles of a hydra, would have the greatest capacity to regenerate. This notion of explaining regeneration by appealing to adaptation remains common (Bely and Nyberg, 2010).

With these three competing interpretations in mind, Thomas Hunt Morgan, the American biologist who was awarded the 1933 Nobel Prize in Physiology or Medicine for his work on genetics, studied regeneration in earthworms, planarians and hydra (Maienschein, 1991). In a book called *Regeneration*, published in 1901, Morgan asked a number of important questions: for example, are existing body parts reshaped during regeneration to become new body parts, or are the new body parts made from new cells and tissue? Morgan concluded that understanding regeneration was one of the outstanding challenges in biology at the time, and that a better explanation of regeneration would lead to a more complete understanding of many other biological processes (Sunderland, 2010). A century later, many of the questions asked by Morgan are still considered important for understanding life (Sánchez Alvarado, 2006).

Lately, the study of regeneration in individual organisms has focused on the idea that certain cells, such as stem cells, have unique regenerative capacities, which could have important applications in medicine. However, there is much that we do not fully understand about stem cells, including how their behavior depends on their environment, and how the effect of the environment varies when we look at different species. As such, stem cells are currently the focus of much basic and translational research, and are also of considerable interest to philosophers of science (Laplane, 2016; Laplane and Solary, 2019).

Before we attempt to develop an overarching understanding or theory of regeneration, we first need to understand what is known about regeneration at the level of individual organisms, ecosystems and microbial communities

## Searching for a broader theory of regeneration

As mentioned above, we are interested in the possibility of finding an overarching understanding or theory that can explain how regeneration works at all levels, from individual organisms through to microbial communities and ecosystems, and this is likely to require us to think carefully about the basic units and mechanisms involved in regeneration.

This should not come as a surprise: at the level of individual organisms, traditional theories of regeneration view cells as the basic unit involved in the detection of damage and in repair, but the properties of a cell often depend on its environment or the system in which it is embedded. Moreover, an individual organism is a system of interacting genes, cells, microbes, chemical influences and other environmental forces. A microbial community also changes over time, with some species of microbes being replaced with other species (sometimes in a way that has an impact on human health). Likewise, an ecosystem consists of microbes, animals and plants, and is also shaped by interactions with humans and other factors.

An important question is: what is regenerating in the case of ecosystems and microbial communities? Consider a forest undergoing regeneration after being damaged by fire: we might expect any species lost to the fire to be replaced by the same species, but research has shown that regeneration often results in the restored ecosystem containing species that were not present before it was damaged (Johnstone et al., 2016; National Park Service, 2017). On the other hand, the use of certain antibiotics can decrease the microbial diversity in the gut, even when the community function is preserved or restored (Antonopoulos et al., 2009).

Developing a theory of regeneration that works across these broad scales of living systems will require us to understand the meaning of regeneration in current literature and research, and to identify the essential components of the process and their interactions within different systems. We will need to address the units that detect injury, begin the repair process, rebuild what has been lost or damaged, and stop regeneration. We need to bear in mind that such units may appear very different at the various scales of life. For instance, in individual organisms, cell signaling molecules may indicate that an area needs to be repaired, whereas in ecosystems, the signal may be a sudden alteration in nitrogen availability. Once we understand such essential components of the regenerative process, we can begin to compare and refine our knowledge of how they work across diverse living systems and construct a theory that is consistent with the evidence.

The process of how living systems detect and respond to damage and injury is a good place to start asking questions about the principles that govern living systems. Making progress in this endeavor will require the combined efforts of scientists, philosophers and historians alike.

## Note

This Feature Article is part of the Philosophy of Biology collection.
